# Evaluation of the predictive value of scoring systems in diagnosis of acute appendicitis: a comparative prospective study

**DOI:** 10.1007/s00423-026-03976-w

**Published:** 2026-02-18

**Authors:** Ayman Shemes, Amr A. Elgharib, Ahmed Elghrieb, Mohamed Shetiwy, Mahmoud A. Aziz, Shady Elzeftawy

**Affiliations:** 1 Department of General Surgery, Faculty of Medicine, Mansoura University, Mansoura, Egypt; 2 Intern Doctor, Faculty of Medicine, Mansoura University, Mansoura, Egypt

**Keywords:** Acute appendicitis, Acute abdomen, Alvarado score, Appendicitis inflammatory response score (AIR), The RIPASA (Raja isteri pengiran anak saleha appendicitis) score and AAS (Adult appendicitis score)

## Abstract

**Background:**

Acute Appendicitis stands as the leading cause of acute abdominal pain necessitating surgical intervention in the world. The initial clinical assessment of patients suspected of having acute appendicitis remains crucial. The aim is to quickly confirm or rule out the diagnosis to minimize delays, avoid unnecessary surgeries.

**Aim and objectives:**

The aim of our study is to evaluate the predictive value of multiple scoring systems for Diagnosis of acute appendicitis cases with correlation between scores prediction results and surgical interventions results.

**Patients and methods:**

This a prospective study carried out in Mansoura university hospitals and Mansoura emergency hospital in the period between December 2024 and June 2025. Patients included in the study were admitted at emergency department of General Surgery at Mansoura University Hospital. The study was explained in details to the whole patients sharing in the study and a written informed consent was taken from all the patients.

All patient presented with suspected acute appendicitis were subjected to four scores with comparing between them : Alvarado score, Appendicitis Inflammatory Response score (AIR), The RIPASA (Raja Isteri Pengiran Anak Saleha Appendicitis) score and AAS (Adult Appendicitis Score).

**Results:**

ROC curve analysis demonstrated variable diagnostic performance among the evaluated scoring systems for acute appendicitis. The Adult Appendicitis Score (AAS) showed the highest discriminatory ability with an AUC of 0.988 (*p* = 0.001), followed by the Appendicitis Inflammatory Response (AIR) score (AUC = 0.920, *p* = 0.005) and the RIPASA score (AUC = 0.825, *p* = 0.03). The Alvarado score demonstrated comparatively lower accuracy (AUC = 0.715, *p* = 0.155). At their respective optimal cutoff points, AAS and AIR achieved high sensitivity and overall accuracy, with AAS demonstrating an apparent specificity of 100%. These findings suggest superior diagnostic performance of AAS and AIR compared with Alvarado and RIPASA in the studied population.

**Conclusion:**

Among the evaluated clinical scoring systems, the Adult Appendicitis Score (AAS) and the Appendicitis Inflammatory Response (AIR) score demonstrated the highest diagnostic accuracy for acute appendicitis, with AAS showing the best overall performance. These scoring systems may represent reliable tools for supporting clinical decision-making and improving diagnostic confidence, potentially reducing unnecessary imaging or negative appendectomies. However, further validation in larger, more diverse cohorts is required before firm recommendations can be made.

## Introduction

Acute Appendicitis stands as the leading cause of acute abdominal pain necessitating surgical intervention in the world [1]. The initial clinical assessment of patients suspected of having acute appendicitis remains crucial. The aim is to quickly confirm or rule out the diagnosis to minimize delays, avoid unnecessary surgeries (where the appendix is found to be normal), prevent appendiceal perforation, and reduce hospital costs [2]. Both confirming and excluding the diagnosis of Acute appendicitis are important for better and cost-effective management. Physical or laboratory findings can’t be used alone to predict or exclude a diagnosis of AA [3]. There are various clinical scoring systems combining physical findings and/or laboratory data have been adopted in regard to this issue such as the Alvarado score, Appendicitis Inflammatory Response score (AIR), The RIPASA (Raja Isteri Pengiran Anak Saleha Appendicitis) score and AAS (Adult Appendicitis Score) [4, 5].

### Aim of the work

The aim of our study is to evaluate the predictive value of multiple scoring systems for Diagnosis of acute appendicitis cases with correlation between scores prediction results and surgical interventions results.

### Patients and methods

This a prospective study carried out in Mansoura university hospitals and Mansoura emergency hospital in the period between December 2024 and June 2025. Patients included in the study were admitted at emergency department of General Surgery at Mansoura University Hospital. The study was explained in details to the whole patients sharing in the study and a written informed consent was taken from all the patients.

### Sample size

Sample size calculation was based on Diagnostic Efficacy of different scores in differentang acute appendicities cases. Depending on sensitivity 89%, precision 0.10 and confidence level 95%, expected revalence of 66% then total sample size will be 57.

All patient presented with suspected acute appendicitis were subjected to four scores with comparing between them:

1. Alvarado score.
2. Appendicitis Inflammatory Response score (AIR).
3. The RIPASA (Raja Isteri Pengiran Anak Saleha Appendicitis) score.
4. AAS (Adult Appendicitis Score).

#### Inclusion criteria

- 1 – Patient age > 16 years with acute appendicitis
- 2 – All patients accepted to participate in the study.

#### Exclusion criteria

- 1- Patients whose age less than 16 years old.
- 2- patients presented with septic shock.
- 3- Patients who had a pre-operative abdominal CT scan for the diagnosis.

### Gold-standard for diagnosing or excluding the condition

Histopathological examination of the surgical biopsy specimen of the appendix was used a gold-standard reference for either confirming or excluding acute appendicitis. (Table 1, 2, 3 and 4).

**Table 1:** Alvarado score [6]

Criteria	Range	Interpretation
- Migratory right iliac fossa pain (1) - Anorexia (1) - Nausea and vomiting (1) - Right iliac fossa tenderness (2) - Rebound tenderness (1) - Elevated temperature (1) - Leukocytosis (2) - Shift to the left of neutrophils (1)	**0–10**	0–4: Low probability of appendicitis 5–6: Possible appendicitis 7–8: Probable Appendicitis 9–10: Very probable appendicitis

**Table 2:** AIR score [7]

Criteria	Range	Interpretation
- Vomiting (1) - Right lower quadrant pain (1) - Rebound tenderness (light = 1, medium = 2, strong = 3) - Fever (1) - Polymorphonuclear leukocytes (< 70% = 0, 70- 84% = 1, ≥ 85% = 2) - White blood cell count (< 10 × 109/L = 0, 10- 14.9 × 109/L = 1, ≥ 15 × 109/L = 2) -Serum C-reactive protein (< 10 mg/dL = 0, 10–49 mg/dL = 1, > 50 mg/dL = 2)	**0–12**	0–4: Low probability of appendicitis. 5–8: Moderate probability 9–12: High probability

**Table 3:** RIPASA score [8]

Criteria	Range	Interpretation
- Male (1) - Age < 40 years (1) - Migratory right iliac fossa pain (0.5) - Anorexia (1) - Nausea and vomiting (1) - Right iliac fossa pain (1) - Right iliac fossa tenderness (1) - Rebound tenderness (1) - Guarding (2) - Rovsing’s sign (2) - Fever (1) - Raised white blood cell count (1) - Negative urinalysis (1)	**0–16.5.5**	< 5: Low probability of Appendicitis 5–7: Low intermediate probability 7.5–11.5: high intermedite probability > 12: High probability of Appendicitis

**Table 4:** AAS score [9]

AAS score Criteria	Score	Interpretation
- Pain in RLQ (2)		*Adult Appendicitis Score (AAS): score* ⩽*10 low risk of appendicitis*, *score 11–15 intermediate risk of appendicitis*,* and score* ⩾*16 high risk of* *appendicitis*
- Pain relocation (2)		
- RLQ tenderness Women aged 16–49 years (1) All other patients (3)		
Guarding Mild (2) Moderate or severe (4)		
- Blood leukocyte count (×109) ⩾7.2 and < 10.9 (1) ⩾10.9 and < 14.0 (2) ⩾14.0 (3)		
- Proportion of neutrophils (%) ⩾62 and < 75 (2) ⩾75 and < 83 (3) ⩾83 (4)		
CRP (mg/L), symptoms < 24 h ⩾4 and < 11 (2) ⩾11 and < 25 (3) ⩾25 and < 83 (5) ⩾83 (1)		
CRP (mg/L), symptoms > 24 h ⩾12 and < 53 (2) ⩾53 and < 152 (2) ⩾152 (1)		

## Method

All the patients were subjected to full detailed history taking and proper general and local examinations.

- Abdominal ultrasound will be done to all patients before admission.
- Laboratory investigations (Complete blood Picture (CBC), C-Reactive protein, INR, urine analysis, Liver and kidney function test) will be done before admission.
- Pregnancy test in female cases presented with suspected acute appendicitis was done.
- Patient age and sex were recorded for all participants and incorporated into score calculation where applicable (e.g., RIPASA and AAS). As demographic variables were not evaluated as independent outcome predictors, a separate demographic summary table was not included.

All patient presented with suspected acute appendicitis were subjected to four scores with comparing between them:

1. Alvarado score.
2. Appendicitis Inflammatory Response score (AIR).
3. The RIPASA (Raja Isteri Pengiran Anak Saleha Appendicitis) score.
4. AAS (Adult Appendicitis Score).

The Alvarado, Appendicitis Inflammatory Response (AIR), RIPASA, and Adult Appendicitis Score (AAS) were selected for evaluation due to their widespread clinical use, ease of bedside application, and reliance on routinely available clinical and laboratory parameters.

### Statistical analysis and data interpretation

Data analysis was performed by SPSS software, version 26 (SPSS Inc., PASW statistics for windows version 26. Chicago: SPSS Inc.). Quantitative data were described using mean ± Standard deviation for normally distributed data after testing normality using Kolmogrov-Smirnov test. Significance of the obtained results was judged at the (0.05) level.

- McNemar test & Marginal Homogenity test (Stewart Maxwell) was used to Student t test was used to compare 2 independent groups for normally distributed data.
- The Spearman’s rank-order correlation is used to determine the strength and direction of a linear relationship between two non-normally distributed continuous variables and/or ordinal variables.
- Receiver operating characteristics curve (ROC curve) was used to calculate validity (sensitivity & specificity) of continuous variables with calculation of best cut off point.Predictive values and accuracy are assessed using cross tabulation.

## Results

In this study, four clinical scoring systems: Alvarado, AIR, RIPASA, and AAS, were evaluated for their diagnostic accuracy in cases of suspected acute appendicitis. Receiver operating characteristic (ROC) curve analysis demonstrated significant differences in the diagnostic performance of the evaluated scoring systems for acute appendicitis. The Appendicitis Assessment Score (AAS) showed the highest discriminatory ability with an area under the curve (AUC) of 0.988 (95% CI: 0.961–1.0, *p* = 0.001), followed by the Appendicitis Inflammatory Response (AIR) score (AUC = 0.920, 95% CI: 0.790–1.0, *p* = 0.005) and the RIPASA score (AUC = 0.825, 95% CI: 0.605–1.0, *p* = 0.03). The Alvarado score demonstrated lower diagnostic performance (AUC = 0.715, 95% CI: 0.337–1.0, *p* = 0.155). At the optimal cutoff values (≥ 14 for AAS, ≥ 6 for AIR, ≥ 12 for RIPASA, and ≥ 7 for Alvarado), AAS achieved a sensitivity of 94.3% and a specificity of 100%, while AIR demonstrated the highest sensitivity (98.1%) with a specificity of 75.0%. Both RIPASA and Alvarado scores showed sensitivities of 86.8% and specificities of 75.0%. Positive predictive values were high across all scoring systems (97.9–100%), whereas negative predictive values ranged from 30.0% to 75.0%. Overall diagnostic accuracy was highest for AIR (96.5%) and AAS (94.7%), confirming their superior performance compared with RIPASA (94.7%) and Alvarado (85.9%) in the studied cohort.

**Fig. 1:**
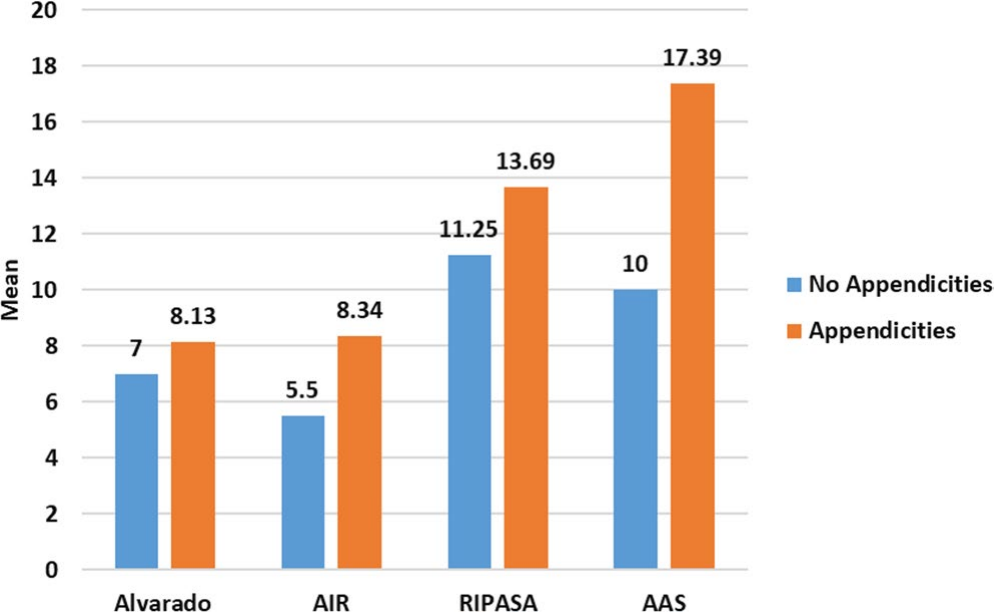
Comparison of different studied scores in determining cases with acute appendicitis in the present study

In details, Table (5) and Fig. (1) present a comparative analysis of mean scores for the Alvarado, AIR, RIPASA, and AAS systems among patients with and without confirmed diagnosis of acute appendicitis. The data show that mean scores were consistently higher in patients with appendicitis across all scoring systems, with AIR, RIPASA, and AAS demonstrating statistically significant differences (*p* = 0.002, *p* = 0.017, and *p* = 0.001, respectively), indicating their superior discriminatory ability. Alvarado showed a non-significant trend toward higher scores in appendicitis cases (*p* = 0.09).

**Table 5:** Comparison of different studied scores in determining cases with acute appendicitis in the present study

	Appendicitis		*p* value
	-VE (*N* = 4)	+VE (*N* = 53)	
Alvarado	7 ± 2	8.13 ± 1.21	t = 1.72 *p* = 0.09
AIR	5.50 ± 1.0	8.34 ± 1.74	t = 3.20 *p* = 0.002*
RIPASA	11.25 ± 1.71	13.69 ± 1.92	t = 2.47 *p* = 0.017*
AAS	10 ± 2.0	17.39 ± 2.44	t = 5.91 *p* = 0.001*

t = Student t test, *statistically significant, data expressed as mean ± SD

**Fig. 2:**
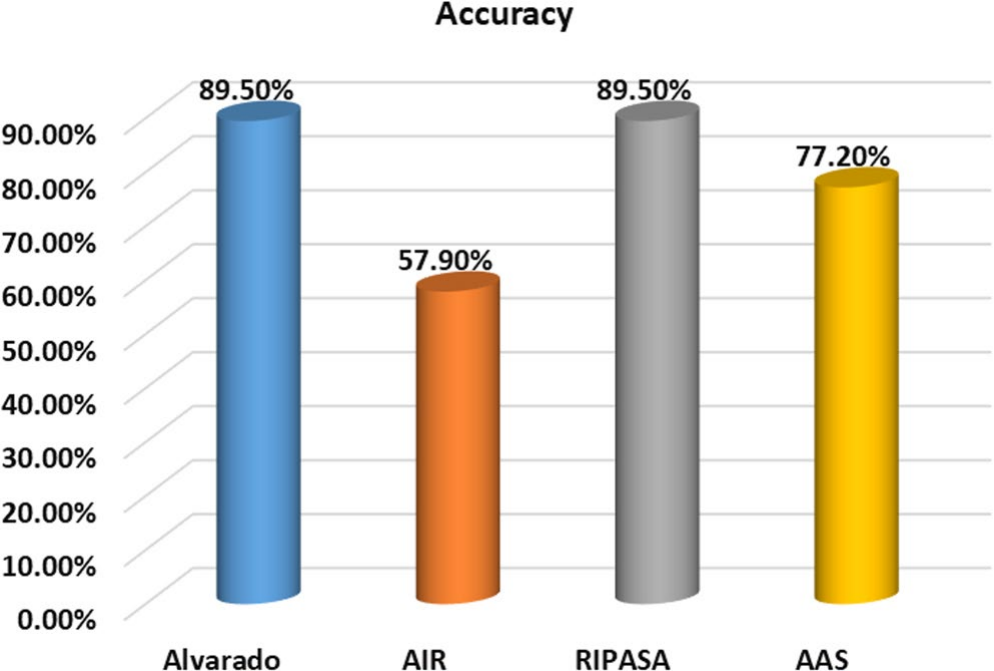
Comparison of studied scores according to reference values *published* in differentiating cases with acute appendicitis [10–12]

Table (6) and Fig. (2) illustrate the sensitivity, specificity, positive predictive value (PPV), negative predictive value (NPV), and accuracy of each scoring system according to reference cutoff values from published literature. All scoring systems demonstrated perfect specificity and PPV (100%), reflecting a strong ability to correctly identify true cases when positive. However, this inflated specificity can be attributed to the limited sample size which presents a major limitation that prevents definitive conclusions. Sensitivity varied, with Alvarado and RIPASA showing higher sensitivity (88.7%) compared to AAS (75.5%) and AIR (54.7%). However, all systems showed low NPV, indicating limited reliability in ruling out appendicitis when scores are low. These findings emphasize that while the studied scores effectively confirm the diagnosis of appendicitis, they are less reliable to exclude the diagnosis.

**Table 6:** Comparison of studied scores according to reference values *published* in differentiating cases with acute appendicitis [10–12]

	Sensitivity	Specificity	PPV	NPV	Accuracy
Alvarado	88.7%	100%	100%	40%	89.5%
AIR	54.7%	100%	100%	14.3%	57.9%
RIPASA	88.7%	100%	100%	40%	89.5%
AAS	75.5%	100%	100%	23.5%	77.2%

PPV: Positive predictive value NPV: Negative predictive value

**Fig. 3:**
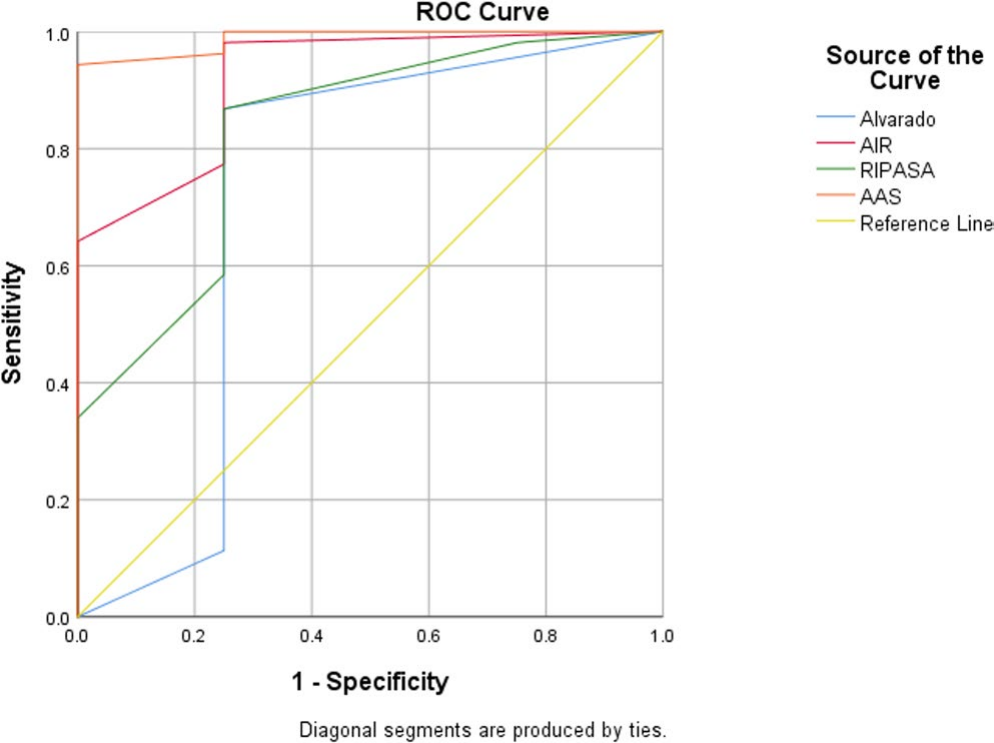
ROC curve of *studied* scores in differentiating cases with appendicitis in the present study

Table (7) and Fig. (3) display the ROC curve analysis results of the current study, showing the area under the curve (AUC), optimal cutoff points, sensitivity, specificity, PPV, NPV, and accuracy for each scoring system in diagnosing acute appendicitis. The AAS demonstrated the highest AUC (0.988, *p* = 0.001), indicating excellent diagnostic performance, followed by AIR (AUC = 0.920, *p* = 0.005) and RIPASA (AUC = 0.825, *p* = 0.03). Alvarado exhibited a lower AUC (0.715, *p* = 0.155), suggesting comparatively lower discriminatory power. These findings confirm that AAS and AIR scores provide superior diagnostic accuracy and reliability in identifying cases of acute appendicitis, with AAS particularly excelling in clinical performance.

**Table 7:** ROC curve of studied scores in differentiating cases with appendicitis in the present study

	AUC (95%CI)	*P* value	Cut off point	Sensitivity	Specificity	PPV	NPV	Accuracy
Alvarado	0.715 (0.337–1.0.337.0)	0.155	≥ 7	86.8%	75.0%	97.9%	30%	85.9%
AIR	0.920(0.790–1.0.790.0)	0.005*	≥ 6	98.1%	75.0%	98.1%	75.0%	96.5%
RIPASA	0.825 (0.605–1.0.605.0)	0.03*	≥ 12	86.8%	75.0%	97.9%	30.0%	94.7%
AAS	0.988 (0.961–1.0.961.0)	0.001*	≥ 14	94.3%	100.0%	100.0%	57.1%	94.7%

PPV: Positive predictive value NPV: Negative predictive value

**Fig. 4:**
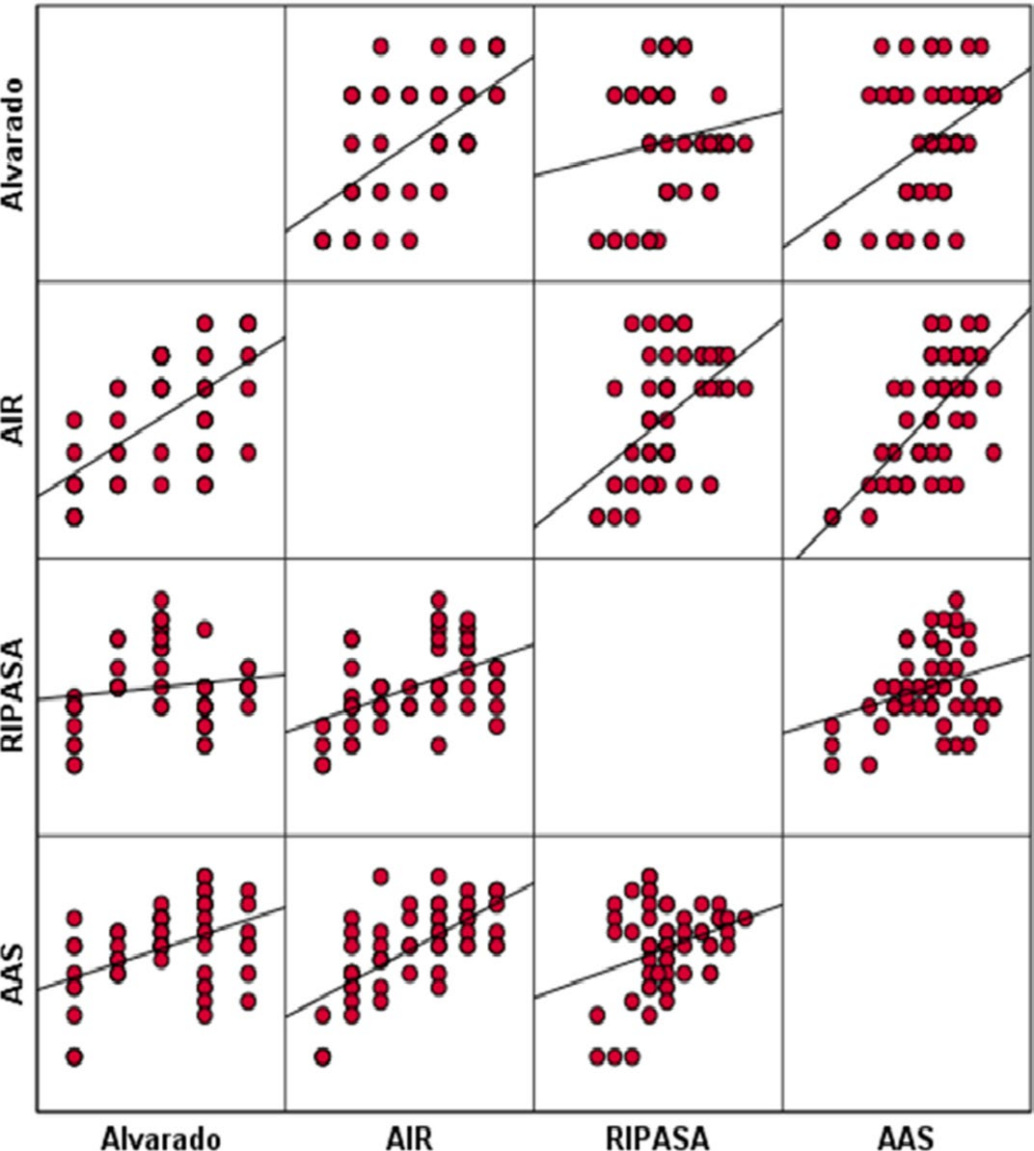
Scatter diagram showing correlation between different scores for differentiating appendicitis

Table (8) and Fig. (4) show the correlation matrix and scatter plots illustrating the relationships between the different scoring systems in diagnosing appendicitis. Significant positive correlations were observed, particularly between AAS and AIR (*r* = 0.683, *p* < 0.05), indicating a strong association in their scoring patterns among cases studied. Alvarado showed moderate correlations with AIR (*r* = 0.605, *p* < 0.05) and AAS (*r* = 0.441, *p* < 0.05), while RIPASA demonstrated lower but still significant correlations with AIR (*r* = 0.488, *p* < 0.05) and AAS (*r* = 0.309, *p* < 0.05). These correlations support the concurrent validity of these scoring systems and suggest that while the scores align in trends, AAS and AIR may provide complementary value in the clinical assessment of suspected appendicitis. While most scoring systems demonstrated significant positive correlations, particularly between AAS and AIR. RIPASA showed a weak and non-significant correlation with Alvarado, indicating partial rather than complete alignment among the evaluated scores.

**Table 8:** Correlation matrix between different scores for differentiating appendicitis

	Alvarado	AIR	RIPASA	AAS
Alvarado	1			
AIR	0.605*	1		
RIPASA	0.145	0.488*	1	
AAS	0.441*	0.683*	0.309*	1.0

Data assessed by correlation coefficient, *statistically significant

## Discussion

Acute appendicitis (AA), the inflammation of the appendix, remains one of the most common causes of acute abdominal pain presenting to emergency departments (EDs), with a lifetime incidence of approximately 7% in the general population [13]. Despite its frequency, the diagnosis of AA continues to pose a clinical challenge due to atypical presentations, particularly among young adults, elderly patients, and females. Gynecological and urogenital conditions may mimic appendicitis, leading to diagnostic uncertainty and potential delays in management [14]. To improve diagnostic accuracy and reduce negative appendectomy rates, several clinical scoring systems have been developed. Among these, the Alvarado score- based on clinical symptoms, physical findings, and laboratory parameters- has been the most widely used. However, previous studies have demonstrated reduced diagnostic accuracy of the Alvarado score, particularly in Eastern populations [15]. The Acute Inflammatory Response (AIR) score was introduced to enhance diagnostic discrimination by incorporating C-reactive protein (CRP) and detailed inflammatory markers alongside clinical criteria [16]. More recently, the Raja Isteri Pengiran Anak Saleha Appendicitis (RIPASA) score was developed, integrating 14 clinical and laboratory parameters, and has been shown to outperform the Alvarado score in several Asian populations with different ethnic and dietary characteristics [17]. In addition, the Adult Appendicitis Score (AAS) has emerged as a comprehensive scoring system designed to improve risk stratification and clinical decision-making. Accordingly, the present study aimed to evaluate and compare the diagnostic performance of multiple appendicitis scoring systems and to assess the correlation between score predictions and surgical outcomes.

The study included 57 patients presenting with suspected acute appendicitis to the Emergency Department of General Surgery at Mansoura University Hospital. Histopathological examination confirmed acute appendicitis in 53 patients, while 4 cases were normal, yielding a negative appendectomy rate of 7%. Comparative analysis revealed that mean scores for all evaluated systems were higher in patients with confirmed appendicitis. Statistically significant differences were observed for AIR, RIPASA, and AAS scores (*p* = 0.002, *p* = 0.017, and *p* = 0.001, respectively), highlighting their superior discriminatory ability, whereas the Alvarado score demonstrated only a non-significant trend (*p* = 0.09). The diagnostic performance of the scoring systems in the present study was variable rather than uniformly perfect. At the optimal cutoff points derived from ROC analysis, AIR demonstrated the highest sensitivity (98.1%) with a specificity of 75.0%, while AAS showed high sensitivity (94.3%) and an apparent specificity of 100%. RIPASA and Alvarado demonstrated moderate sensitivity (86.8% for both) and specificity (75.0% for both). Positive predictive values were high across all scoring systems, reflecting the high prevalence of appendicitis in the study cohort; however, negative predictive values were relatively low, particularly for Alvarado and RIPASA scores, indicating limited reliability in excluding appendicitis when scores were below the cutoff. These findings suggest that while these scoring systems are useful for confirming the diagnosis of AA, caution is warranted when using them to rule out the disease. ROC curve analysis further supported these findings, with AAS demonstrating the highest area under the curve (AUC = 0.988, *p* = 0.001), indicating excellent diagnostic performance. This was followed by the AIR score (AUC = 0.920, *p* = 0.005) and RIPASA score (AUC = 0.825, *p* = 0.03), while the Alvarado score exhibited lower discriminatory ability (AUC = 0.715, *p* = 0.155). These results confirm that AAS and AIR provide superior diagnostic accuracy compared with Alvarado and RIPASA in the present cohort. In agreement with the current findings, Shuaib et al. (2017) reported superior sensitivity and specificity of the RIPASA score compared with the modified Alvarado score, along with better negative predictive value and diagnostic accuracy [18]. Similarly, Chong et al. (2010) documented sensitivity and specificity values of 88% and 67%, respectively, for RIPASA in an Asian population, with a negative appendectomy rate of 22.9% [19]. Nanjundaiah et al. (2014) also demonstrated higher sensitivity for RIPASA compared with Alvarado, although specificity varied between studies [20]. Conversely, Ak et al. (2020) reported higher diagnostic accuracy for RIPASA compared with AIR and Alvarado scores, with AIR demonstrating the highest sensitivity but lower specificity [14], highlighting the influence of population characteristics and study design on scoring system performance. Correlation analysis in the present study demonstrated significant positive relationships among the evaluated scoring systems, particularly between AAS and AIR (*r* = 0.683, *p* < 0.05), indicating strong concordance in their assessment patterns. Moderate correlations were observed between Alvarado and both AIR and AAS, while RIPASA showed weaker but statistically significant correlations. These findings support the concurrent validity of the scoring systems and suggest that AAS and AIR may offer complementary diagnostic value in clinical practice. Importantly, the apparent 100% specificity observed for AAS in the present study should be interpreted with caution. This finding is most likely attributable to the limited sample size and the small number of non-appendicitis cases, which may have resulted in an overestimation of specificity. Consequently, while AAS and AIR appear highly promising tools for the diagnosis of acute appendicitis, larger prospective studies are required to validate these results and establish definitive conclusions regarding their true diagnostic performance.

## Conclusion

Among the evaluated clinical scoring systems, the Adult Appendicitis Score (AAS) and the Appendicitis Inflammatory Response (AIR) score demonstrated the highest diagnostic accuracy for acute appendicitis, with AAS showing the best overall performance. These scoring systems may represent reliable tools for supporting clinical decision-making and improving diagnostic confidence, potentially reducing unnecessary imaging or negative appendectomies. However, further validation in larger, more diverse cohorts is required before firm recommendations can be made.

### Limitations

This study has an important limitation related to its relatively small sample size. The limited number of cases likely contributed to an overestimation of specificity, particularly for the AAS, which demonstrated an apparent specificity of 100%. This inflated specificity restricts the ability to draw definitive conclusions regarding the true diagnostic performance of the scoring systems. Larger prospective studies are therefore necessary to confirm these findings and ensure their generalizability. In addition, our study was the one-centered design.

## Data Availability

No datasets were generated or analysed during the current study.

## References

[1] Gore RM, Levine MS (2008) Textbook of Gastrointestinal radiology. Saunders/Elsevier

[2] Bhangu A, Søreide K, Di Saverio S, Assarsson JH, Drake FT (2015) Acute appendicitis: modern Understanding of pathogenesis, diagnosis, and management. Lancet 386(10000):1278–1287 26460662 10.1016/S0140-6736(15)00275-5

[3] Bom WJ, Scheijmans JC, Salminen P, Boermeester MA (2021) Diagnosis of uncomplicated and complicated appendicitis in adults. Scand J Surg 110(2):170–179 33851877 10.1177/14574969211008330 PMC8258714

[4] Song H, Lee S, Park JH, Kim HY, Min HD, Jeon JJ, Lee KH, LOCAT Group (2021) Can patient triaging with clinical scoring systems reduce CT use in adolescents and young adults suspected of having appendicitis? Radiology 300(2):350–358 34003054 10.1148/radiol.2021203884

[5] Malik MU, Connelly TM, Awan F, Pretorius F, Fiuza-Castineira C, El Faedy O, Balfe P (2017) The ripasa score is sensitive and specific for the diagnosis of acute appendicitis in a western population. Int J Colorectal Dis 32(4):491–7 27981378 10.1007/s00384-016-2713-4

[6] Sobnach S, Ede C, Van Der Linde G, Klopper J, Thomson S, Bhyat A, Kahn D (2018) A retrospective evaluation of the Modified Alvarado Score for the diagnosis of acute appendicitis in HIV-infected patients. Eur J Trauma Emerg Surg 44(2):259–63 28573428 10.1007/s00068-017-0804-8

[7] Deiters A, Drozd A, Parikh P, Markert R, Shim JK (2019) Use of the Alvarado score in elderly patients with complicated and uncomplicated appendicitis. Am Surg 85(4):397–402 31043201

[8] Butt MQ, Chatha SS, Ghumman AQ, Farooq M (2014) Ripasa score: a new diagnostic score for diagnosis of acute appendicitis. J Coll Physicians Surg Pak 24(12):894–897 25523723

[9] Sammalkorpi HE, Mentula P, Savolainen H, Leppäniemi A (2017) The introduction of adult appendicitis score reduced negative appendectomy rate. Scand J Surg 106(3):196–201 28737110 10.1177/1457496916683099

[10] Ghali MS, Hasan S, Al-Yahri O, Mansor S, Al-Tarakji M, Obaid M, Shah AA, Shehata MS, Singh R, Al-Zoubi RM, Zarour A (2023) Adult appendicitis score versus Alvarado score: a comparative study in the diagnosis of acute appendicitis. Surg Open Sci 14:96–102 37577253 10.1016/j.sopen.2023.07.007 PMC10413131

[11] Von-Muehlen B, Franzon O, Beduschi MG, Kruel N, Lupselo D (2015) AIR score assessment for acute appendicitis. ABCD. Arquivos Brasileiros de Cirurgia Digestiva (São Paulo) 28(3):171–3 10.1590/S0102-67202015000300006 PMC4737355 26537139

[12] Pattiiha AM, Selomo PA, Faruk M (2022) Comparison of the RIPASA and Labeda scoring systems to assess the morphological severity of acute appendicitis. Open Access Maced J Med Sci 10(B):1996–1999

[13] Echevarria S, Rauf F, Hussain N, Zaka H, Ahsan N, Broomfield A, Akbar A, Khawaja UA, Farwa UE (2023) Typical and atypical presentations of appendicitis and their implications for diagnosis and treatment: a literature review. Cureus. ;15(4) 10.7759/cureus.37024 PMC10152406 37143626

[14] Ak R, Doğanay F, Unal Akoğlu E, Akoğlu H, Uçar AB, Kurt E, Arslan Turan C, Onur O (2020) Predictive value of scoring systems for the diagnosis of acute appendicitis in emergency department patients: is there an accurate one? Hong Kong J Emerg Med 27(5):262–269

[15] RAMEZ MW, AKHNOKH SG, Kamel SF (2020) Evaluation of Alvarado score in the diagnosis of acute appendicitis. Med J Cairo Univ 88(September):1663–1672

[16] Noori IF, Jabbar AS, Noori AF (2023) Clinical scores (Alvarado and AIR scores) versus imaging (ultrasound and CT scan) in the diagnosis of equivocal cases of acute appendicitis: a randomized controlled study. Ann Med Surg 85(4):676–683 10.1097/MS9.0000000000000270 PMC10129239 37113930

[17] Mumtaz H, Sree GS, Vakkalagadda NP, Anne KK, Jabeen S, Mehmood Q, Mehdi Z, Sohail H, Haseeb A, Zafar Y, Saghir S (2022) The RIPASA scoring system: A new era in appendicitis diagnosis. Annals Med Surg 80:104174 10.1016/j.amsu.2022.104174 PMC9422193 36045852

[18] Shuaib A, Shuaib A, Fakhra Z, Marafi B, Alsharaf K, Behbehani A (2017) Evaluation of modified Alvarado scoring system and RIPASA scoring system as diagnostic tools of acute appendicitis. World J Emerg Med 8(4):276 10.5847/wjem.j.1920-8642.2017.04.005 PMC5675968 29123605

[19] Chong CF, Adi MI, Thien A, Suyoi A, Mackie AJ, Tin AS, Tripathi S, Jaman NH, Tan KK, Kok KY, Mathew VV (2010) Development of the RIPASA score: a new appendicitis scoring system for the diagnosis of acute appendicitis. Singapore Med J 51(3):220 20428744

[20] Nanjundaiah N, Mohammed A, Shanbhag V, Ashfaque K, Priya SA (2014) A comparative study of RIPASA score and ALVARADO score in the diagnosis of acute appendicitis. J Clin Diagn Research: JCDR 8(11):NC03 10.7860/JCDR/2014/9055.5170 PMC4290278 25584259

